# Activity of Cinnamaldehyde on Quorum Sensing and Biofilm Susceptibility to Antibiotics in *Pseudomonas aeruginosa*

**DOI:** 10.3390/microorganisms8030455

**Published:** 2020-03-23

**Authors:** Sanjida Halim Topa, Enzo A. Palombo, Peter Kingshott, Linda L. Blackall

**Affiliations:** 1 Department of Chemistry and Biotechnology, Swinburne University of Technology, Hawthorn, VIC 3122, Australia; epalombo@swin.edu.au (E.A.P.); pkingshott@swin.edu.au (P.K.); linda.blackall@unimelb.edu.au (L.L.B.); 2 School of Biosciences, The University of Melbourne, Parkville, VIC 3052, Australia

**Keywords:** quorum sensing, cinnamaldehyde, *Pseudomonas aeruginosa*, subminimum inhibitory concentrations, biofilm

## Abstract

Quorum sensing (QS) plays an important role during infection for the opportunistic human pathogen *Pseudomonas aeruginosa*. Quorum sensing inhibition (QSI) can disrupt this initial event of infection without killing bacterial cells, and thus QS inhibitors have been suggested as novel approaches for anti-infective therapy. Cinnamaldehyde (CAD) is a *P. aeruginosa* biofilm inhibitor and disperser of preformed biofilms. In this study, the combined use of CAD and colistin (COL) revealed a synergistic activity, but this was not the case for CAD combined with carbenicillin, tobramycin (TOB), or erythromycin in checkerboard assays for *P. aeruginosa*. CAD demonstrated QSI activity by repression of the expression of *lasB*, *rhlA* and *pqsA* in GFP reporter assays. Approximately 70% reduction in GFP production was observed with the highest CAD concentration tested in all the QS reporter strains. TOB also showed strong QSI when combined with CAD in reporter assays. Combination treatments revealed an additive activity of CAD with COL and TOB in biofilm inhibition (75.2% and 83.9%, respectively) and preformed biofilm dispersion (~90% for both) when compared to the individual treatments. Therefore, a proposed method to mitigate *P. aeruginosa* infection is a combination therapy of CAD with COL or CAD with TOB as alternatives to current individual drug therapies.

## 1. Introduction

The 20th century is recognized as the “antibiotic era”, which started with the discovery of antibiotics to fight bacterial infections. However, this has been marred by the emergence of multidrug-resistant bacteria [1] which has diminished the efficacy of antibiotic treatments. While the spread of antibiotic resistance is largely due to horizontal gene exchange or the acquisition of mutations associated with resistance, it is increasingly recognized that bacteria can exhibit increased tolerance to antimicrobials when they grow as a biofilm [2,3]. Biofilm formation is associated with the establishment of persistent and chronic infections [4,5]. The role of biofilms in forming chronic, drug-tolerant infections is particularly well known for *Pseudomonas aeruginosa*, which is one of the most studied pathogens for antimicrobial research according to the Infectious Diseases Society of America (IDSA) [6,7].

*P. aeruginosa* is an opportunistic pathogen that employs a number of different pathogenic traits (e.g., biofilm formation) by means of a cell-to-cell communication system, termed quorum sensing (QS) [8]. *P. aeruginosa* utilizes signaling molecules to coordinate the expression of virulence factors, such as elastase and rhamnolipids, as well as genes involved in biofilm formation. The QS system of *P. aeruginosa* is comprised of three hierarchically integrated QS systems, Las, Rhl and PQS, to control the expression of these virulence factors and biofilm genes that contribute to its pathogenicity [9]. One emerging strategy for supplementing the existing antibiotic treatment options is through the disruption of QS to inhibit virulence factor expression instead of inhibiting growth [10,11]. Since growth is not dependent on QS, there is a reduced selection pressure for resistance to develop.

A number of publications have identified natural compounds and their synthetic analogues that interfere with QS and that have been shown to reduce virulence factor expression in vitro or virulence in vivo [10,11,12,13,14,15,16,17]. Some natural foods have compounds with QS inhibitory activity, and such foods might offer a natural prophylaxis against chronic *P. aeruginosa* infections [15,16]. An additional benefit of QSI is that QS-mediated biofilm formation is also associated with increased tolerance to antibiotics. Reduction in *P. aeruginosa* biofilm resistance to antibiotics was previously reported by combining a QS inhibitor (e.g., *N*-(2-pyrimidyl)butanamide) and antibiotics (ciprofloxacin, colistin and tobramycin) [18]. Therefore, reducing antibiotic resistance by QSI could be a practical approach in mitigating future crises of antibiotic resistance. The use of subinhibitory concentrations of macrolides was recently reported as another option for developing novel treatment strategies for *P. aeruginosa* infections [19]. Indeed, it has been shown that QS inhibitors work synergistically with antibiotics [20,21,22]. Thus, the application of QS inhibitors with standard antibiotics could be a promising strategy to attenuate biofilm infections [22,23].

Cinnamaldehyde (CAD) is one of the primary phytoconstituents of cinnamon, with therapeutic potential to act as an antimicrobial agent against *P. aeruginosa* [24,25]. In a previous study, we demonstrated that CAD can disrupt biofilms and other surface colonization phenotypes (e.g., swarming motility) of *P. aeruginosa* [26]. CAD also modulated intracellular signaling processes through decreasing cyclic-di-GMP levels [26], which led us to investigate whether CAD could be used as potential antivirulence compound [26]. A previous study with sublethal concentrations of CAD demonstrated inhibition of QS virulence factors and biofilm formation in *P. fluorescence* [27]. In a recent study [28], subinhibitory levels of CAD downregulated *las* and *rhl* of *P. aeruginosa*. We demonstrated that CAD is capable of interfering in the *P. aeruginosa* Las, Rhl and PQS QS systems, while having no impact on bacterial growth. CAD combined with COL or tobramycin (TOB) inhibited biofilm formation and dispersed preformed biofilms more efficiently than individual treatments. We have also exploited a combined positive effect of CAD and TOB on *P. aeruginosa* QS systems.

## 2. Materials and Methods

### 2.1. Bacterial Strains, Media and Culture Conditions

Bacterial strains tagged with green fluorescent protein (GFP) were used in QS assays with *P. aeruginosa* PAO1. The *lasB::gfp* (ASV) [29], *rhlA::gfp* (ASV) [30] and *pqsA::gfp* (ASV) with Gm^R^ [31] translational reporter fusions were used. Reporter strains were routinely grown overnight in Mueller Hinton Broth (MHB, Oxoid, Thermo Fisher Scientific, VIC, Australia) with 125.6 μM gentamicin (Gm, Sigma-Aldrich, NSW, Australia) at 37 °C with shaking at 180 rpm. For QS assays, reporter strains were grown in ABTGC medium, which is AB minimal medium [32] plus 7.4 µM thiamine, 0.01 M glucose and 0.01 M casamino acids [33]. AB minimal medium consists of 15.1 mM ammonium sulfate, 33.7 mM sodium phosphate dibasic, 22 mM potassium dihydrogen phosphate, 50 mM sodium chloride, 1 mM magnesium chloride hexahydrate, 100 µM calcium chloride dehydrate and 1 µM iron (III) chloride hexahydrate [33,34]. All the chemicals to prepare ABTGC medium were purchased from Sigma-Aldrich, NSW, Australia. For the other experiments in this study, a wild-type *P. aeruginosa* PAO1 [33] was grown in MHB overnight at 37 °C with shaking at 180 rpm.

### 2.2. Determination of Minimum Inhibitory Concentration (MIC) for Antibiotics

MICs of four different classes of antibiotics (polypeptide (colistin), penicillin (carbenicillin), aminoglycoside (tobramycin), and macrolide (erythromycin)) were determined using the broth microdilution method [35]. It was carried in 96-well microtiter plates (Nunc, Thermo Fisher Scientific, VIC, Australia) with an MHB overnight culture of *P. aeruginosa* PAO1 adjusted to OD_600_ of 0.1. Two-fold serial dilutions with MHB achieved final concentrations of antibiotics (Sigma-Aldrich, Singapore): colistin (COL 27.2 to 0.2 µM), carbenicillin (CARB 1353.1 to 10.6 µM), tobramycin (TOB 13.3 to 0.1 µM), and erythromycin (ERY 1395.2 to 10.9 µM). The plate was incubated at 37 °C for 18–20 h with shaking at 180 rpm. The lowest concentration of antibiotics inhibiting growth as observed visually was recorded as the MIC for each antibiotic.

### 2.3. Checkerboard Assay to Test Interaction between CAD and Antibiotics

The aim of the experiment was to evaluate the ability of antibiotics combined with CAD to inhibit the growth of P. aeruginosa PAO1. Four different classes of antibiotics were tested in combination with CAD (Product No: W228613, Sigma-Aldrich, NSW, Australia). Interactions of each antibiotic with CAD was assessed using a checkerboard assay in 96-well microtiter plates (Nunc, Thermo Fisher Scientific, VIC, Australia) (12 columns (1 to 12) × 8 rows (A to H)). For the checkerboard assay, each antibiotic was added to individual wells in the microtiter plate at concentrations ranging from 16 to 0.13 MIC (concentrations decreasing from rows A to H) and CAD was added to individual wells at concentrations representing 16 to 0.13 MIC (concentrations decreasing from columns 1 to 8). Columns 9 and 10 were used for testing antibiotics and CAD alone, respectively. Column 11 was used as positive growth control with *P. aeruginosa* grown in MHB alone, and column 12 was used as sterility control with uninoculated MHB. After preparing each well with the appropriate dilutions of CAD and antibiotics, 100 µL of a *P. aeruginosa* PAO1 overnight MHB culture adjusted to OD_600_ of 0.1 was added to each well, and plates were incubated at 37 °C for 18-20 h with shaking at 180 rpm. The MIC for each compound was the lowest concentration that inhibited bacterial growth. The synergistic interactions were expressed as the fractional inhibitory concentration index (FICI), which is calculated as:

(1) ∑FICI=FIC antibiotic+FIC CAD,

where

(2) FIC antibiotic=MIC of antibiotic in combinationMIC of antibiotic alone,

(3) FIC CAD=MIC of CAD in combinationMIC of CAD alone,

A synergistic effect was defined at an FICI of ≤ 0.5; an indifferent effect at an FICI between 0.5 and ≤ 4 and an antagonistic effect at an FICI > 4. Similar checkerboard assays were followed for all four antibiotics.

### 2.4. Development of Resistance to CAD

CAD was tested for development of resistance in *P. aeruginosa* PAO1 by serial passaging [36]. An overnight bacterial culture adjusted to OD_600_ of 0.1 was amended with different concentrations of CAD (23.6, 11.8 (= MIC), 5.9 and 3 mM). Inoculum without CAD was used as a control. Following 24 h incubation at 37 °C with shaking at 180 rpm, the culture with visible growth in the highest CAD concentration was adjusted to an OD_600_ of 0.1 with MHB and used as the inoculum for the next round of growth with CAD at 23.6, 11.8, 5.9 and 3 mM and the same incubation conditions as before. This was repeated for 23 days. Growth curves of *P. aeruginosa* PAO1 treated with 5.9 mM CAD for 24 h were generated at days 1 and 23 to determine the effect of serial passage. The relative growth rate was calculated by dividing the generation time of the 5.9 mM grown culture at day 1 with the generation time of the 5.9 mM grown culture at day 23 [37]. The generation time was determined as follows:

(4) $$Generation\;time,\;G=\frac{t}{3.3log\;b/B},$$

where t is the time interval in min, B is the OD_600_ at the beginning of the time interval and b is the OD_600_ at the end of the time interval

### 2.5. QS Inhibition Assays with CAD or TOB

Overnight cultures of the QS reporter strains were adjusted to an OD_600_ of 0.1 and added with 100 µL of CAD to achieve 3, 1.5 or 0.8 mM CAD in a 96-well plate. Similarly, 0.9 µM TOB alone and 0.9 µM TOB combined with 1.5 mM CAD were tested to determine their combined effect as QS inhibitors. Cultures with no CAD or antibiotics added were used as growth controls. A proprietary synthetic QS inhibitor named “A7” at 10 µM final concentration (kindly provided by Liang Yang, Singapore Centre for Environmental Life Sciences Engineering (SCELSE), Nanyang Technological University, Singapore) was used as a standard QS inhibitor. Plates were incubated at 37 °C with shaking at 180 rpm for 7 h. GFP fluorescence (excitation wavelength of 485–512 nm and emission wavelength of 520–530 nm) and bacterial cell density (OD_600_) measurements were collected at 1, 3, 5 and 7 h using a FLUOstar Omega microplate reader (BMG Labtech, VIC, Australia). The relative fluorescence units (RFU) were measured by dividing the fluorescence value by the corresponding OD_600_ value. *p* values were calculated using regression analyses in Excel and values ≤0.05 were statistically significant.

### 2.6. Biofilm Inhibitory Assay with CAD and Antibiotics

The biofilm inhibitory activities of CAD and antibiotics were determined using Calgary biofilm devices (CBDs) consisting of a 96-well microtiter plate with 96 pegs on the lid (Thermo Fisher Scientific, VIC, Australia). The sub-MICs of CAD and antibiotics used in this experiment were determined from the results of the checkerboard assay. Volumes of 100 µL of overnight MHB cultures of *P. aeruginosa* PAO1 adjusted to OD_600_ of 0.1 were amended with 100 µL of 1:1 ratios of CAD-COL or CAD-TOB (50 µL of CAD + 50 µL of COL or TOB) to achieve final concentrations of 1.5 mM (CAD), 0.9 µM (COL) and 0.9 µM (TOB). The peg lid was added, and plates were incubated at 37 °C with shaking at 180 rpm for 6 h, which was determined to yield the maximum biofilm on the pegs [26]. Peg lids with adherent biofilms were transferred to a fresh plate base containing phosphate-buffered saline (PBS, 137 mM sodium chloride, 2.7 mM potassium chloride, 10 mM sodium phosphate dibasic and 2 mM potassium dihydrogen phosphate (Sigma-Aldrich, NSW, Australia)) to remove loosely attached bacterial cells. Biofilms on the peg lids were stained in 200 µL of 0.1% aqueous crystal violet (CV) (Sigma-Aldrich, NSW, Australia) for 20 min at 37 °C with shaking (180 rpm), rinsed twice with 200 µL PBS, then placed into 200 µL of ethanol (100%) to solubilize the CV. The OD_570_ of the CV in ethanol solution was determined using an Omega microplate reader. Biofilm formation inhibition of tested compounds was measured using Equation (5).

(5) $$\mathrm{Biofilm}\;\mathrm{formation}\;\mathrm{inhibition}\;\%=\left(\frac{OD\;experimental\;well\;without\;\;compound-OD\;experimental\;well\;with\;\;compound}{OD\;experimental\;well\;without\;compound}\right)\times 100,$$

### 2.7. The Effect of CAD and Antibiotics on Preformed Biofilms

MHB cultures of *P. aeruginosa* PAO1 formed 6 h biofilms on peg lids in CBDs as described above. The preformed biofilms were exposed to 1.5 mM CAD, 0.9 µM COL, 0.9 µM TOB, CAD-COL (1.5 mM and 0.9 µM, respectively) or CAD-TOB (1.5 mM and 0.9 µM, respectively) for 3 h at 37 °C with shaking at 180 rpm. Biofilms were washed and CV stained as above, and the biofilm dispersal activity was determined by Equation (6).

(6) $$\mathrm{Preformed}\;\mathrm{biofilm}\;\mathrm{dispersion}\;\%=\left(\frac{OD\;experimental\;well\;without\;compound-OD\;experimental\;well\;with\;compound}{OD\;experimental\;well\;without\;compound}\right)\times 100,$$

## 3. Results

### 3.1. Synergistic Activity of CAD and Antibiotics against Planktonic Cells of P. aeruginosa

The MICs for all the antibiotics (Table 1) were determined with planktonic cells of *P. aeruginosa*. MICs were then used to establish the checkerboard assay to study interactions between CAD and the antibiotics on *P. aeruginosa* growth (Table 1). 

### 3.2. P. aeruginosa Did Not Develop Resistance to CAD with Serial Passage

**Figure 1:**
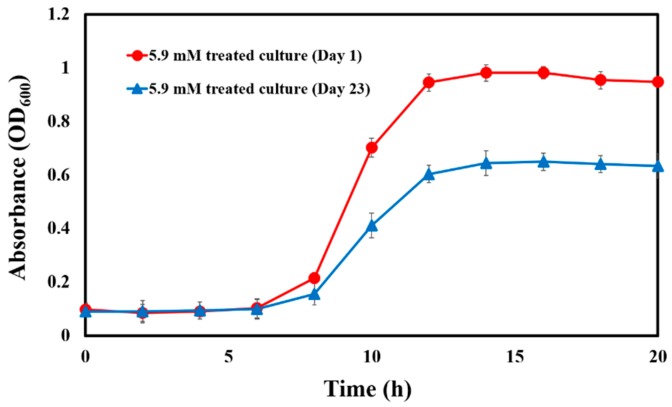
Growth curve of *P. aeruginosa* isolates in the presence of 5.9 mM CAD that were passaged for 1 day (●) or 23 days (▲). The results are the average of three independent experiments in parallel, and error bars indicate ± standard deviations.

Cells serially passaged 23 times in MHB containing CAD at sub-MIC (5.9 mM) did not demonstrate a change in their CAD MIC. However, the resultant culture did not attain the same optical density at the stationary phase of growth (Figure 1), and the calculated log phase generation time increased from 55.5 to 83.4 min. However, there was no change in the lag-to-log phase time (at ~6 h) of the culture grown with CAD on day 1 and on day 23 (Figure 1). Additionally, there was no change in the growth curve of the culture grown without CAD between day 1 and day 23 (data not shown). Thus, the reduction in growth rate with CAD was deemed CAD-specific and not the result of multiple passages of the culture. It could be that the culture evolved over the 23 passages with consistent exposure to CAD, albeit at a sub-MIC level.

### 3.3. QSI Activity of CAD

**Figure 2:**
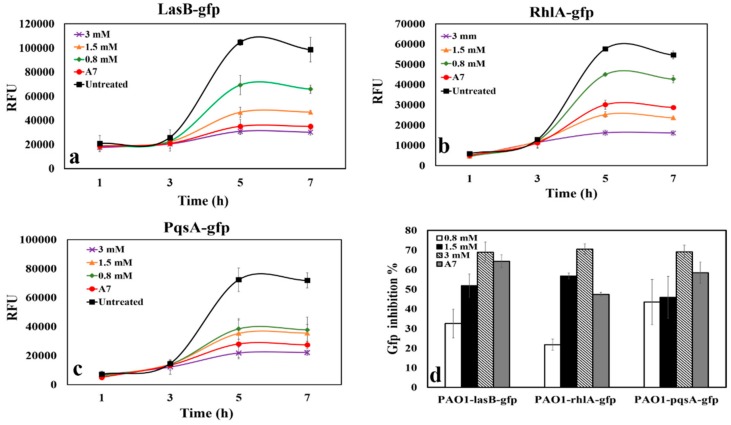
Dose–response curves of CAD incubated with (**a**) *P. aeruginosa* PAO1-*lasB-gfp*; (**b**) *P. aeruginosa* PAO1-*rhlA-gfp* and (**c**) *P. aeruginosa* PAO1-*pqsA-gfp*. ×, 3 mM CAD; ▲, 1.5 mM CAD; ◆, 0.8 mM CAD; ●, A7; ■, untreated. (**d**) Green fluorescent protein (GFP) inhibition percent with varying levels of CAD at 7 h: 0.8 mM CAD (white bars); 1.5 mM CAD (black bars); 3 mM CAD (downward diagonal bars). A7 (gray bars) is a QS inhibitor. Data represent the average of three independent experiments, and error bars indicate ± standard deviations. *p* values of ≤ 0.05. Relative fluorescence units (RFU) were normalized to OD_600_ for all reporter assays.

Elastase (encoded by *lasB*) is a virulence factor that is controlled by LasR and is an indicator of LasR activity [38]. To test the ability of CAD to inhibit LasR, a *P. aeruginosa* PAO1-*lasB-gfp* strain was treated with different concentrations of CAD. At all tested concentrations of CAD, we observed a significant (*p* values of ≤ 0.05) reduction in RFU corresponding to GFP inhibition over 7 h, and there was a decrease in expression of fluorescence after 7 h (data not shown); this reduction was dose dependent (Figure 2a). 

After 7 h incubation, GFP inhibition at the lowest concentration of CAD tested (0.8 mM) was 32.5% of the non-CAD treated control culture. At 1.5 and 3 mM, the inhibition of GFP was 51.9% and 68.9% of the control culture, respectively (Figure 2d). The activity of 3 mM CAD was quite similar to the QSI control compound A7. For all CAD concentrations tested, there was no reduction in biomass as determined by OD_600_ (data not shown), suggesting that the effect on QS was not due to growth inhibition or toxicity of the CAD.

To determine if CAD was specific for the Las system or if it more generally inhibited QS, reporter bioassays for Rhl and PQS were also tested. Incubation of CAD with the *P. aeruginosa* PAO1-*rhlA-gfp* reporter strain (Figure 2b) and the *P. aeruginosa* PAO1-*pqsA-gfp* reporter strain (Figure 2c) showed a dose-dependent inhibition of GFP production. Compared to the inhibition of LasR (32.5%), a slightly weaker inhibition was observed for RhlR (21.8%) (Figure 2d). However, 70.4% inhibition was observed with 3 mM CAD, which is similar to the LasR system. It could be that the lower CAD concentration (0.5 MIC; 0.8 mM) might have higher binding affinity for LasR than for RhlR. Thus, this binding to LasR or RhlR resulted in inhibition of QS expression by disrupting LasR or RhlR activity. CAD also strongly inhibited PQS (43.4%, Figure 2d). In every case, the maximum inhibition attained was not more than 70% at 3 mM CAD (Figure 2d). The highest tested concentration of CAD (3 mM) showed a higher inhibition compared to the synthetic QS inhibitor A7 for all of the QS systems, although the concentration used for A7 (10 µM) was considerably lower than the CAD concentrations that were used here. We exposed a strain of *P. aeruginosa* that constitutively expresses *gfp* to 3 mM CAD and observed no change in RFU [26]. This suggests that CAD does not directly interfere with the stability or fluorescence of GFP.

### 3.4. QS Inhibitory Activity of CAD and TOB

TOB has been demonstrated to inhibit the Rhl QS system in *P. aeruginosa* at sub-MICs [39]. In this study, 1.5 mM CAD and 0.9 µM TOB were assessed to determine their individual (CAD or TOB) and combined (CAD-TOB) QSI effects on the QS GFP reporter strains of *P. aeruginosa*.

**Figure 3:**
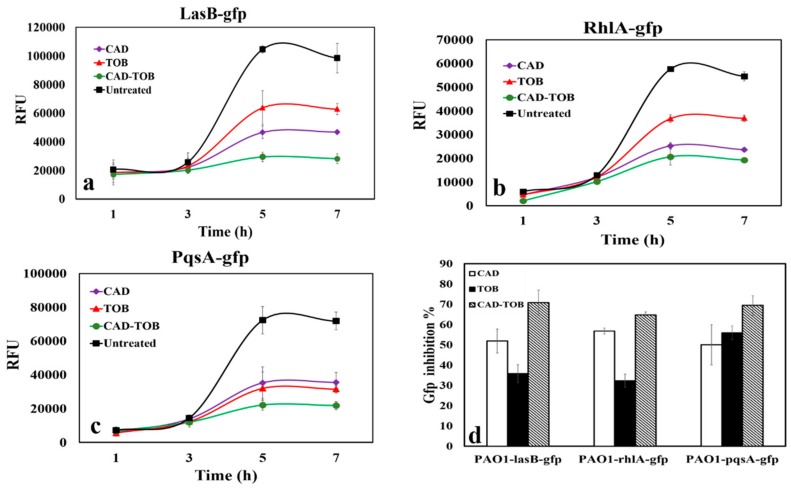
The effect of combined CAD and tobramycin (TOB) treatment on quorum sensing (QS). (**a**) *P. aeruginosa* PAO1-*lasB-gfp*; (**b**) *P. aeruginosa* PAO1-*rhlA-gfp* and (**c**) *P. aeruginosa* PAO1-*pqsA-gfp*. ◆, CAD; ▲, TOB ●; CAD-TOB; ■, untreated. (**d**) GFP inhibition % with CAD and TOB at 7 h. CAD (white bars); TOB (black bars); CAD + TOB (downward diagonal bars). Data represent the average of three independent experiments, and error bars indicate ± standard deviation. *p* values of ≤ 0.05. RFU were normalized to OD_600_ for all reporter assays.

CAD and TOB alone reduced LasR-mediated QS over time, as shown by the reduction in RFU (Figure 3a) and calculated GFP reductions (Figure 3d), compared to the non-CAD treated culture. CAD reduced GFP expression by 51.9% and TOB reduced it by 35.8%, compared to non-CAD treatments, and the combination of CAD and TOB showed a 70.7% GFP reduction in expression (Figure 3d). The combination of CAD and TOB was also assessed for the effects on *rhlA* and *pqsA* as determined by GFP expression (Figure 3b,c). Treatment with CAD and TOB alone reduced RhlR controlled GFP expression by 56.7% and 32.2%, respectively (Figure 3d). CAD and TOB alone reduced PQS by 50% and 55.9%, respectively (Figure 3d). The combination of CAD-TOB repressed RhlR and PQS by 64.7% and 69.4% (Figure 3d), respectively, which was higher than either compound alone. The CAD and TOB concentrations used for this study had no impact on the growth of *P. aeruginosa* PAO1 (data not shown). These results demonstrate that CAD and TOB in combination have an additive effect in disrupting Las, Rhl and PQS QS systems.

### 3.5. Biofilm Inhibition by CAD, COL and TOB

**Figure 4:**
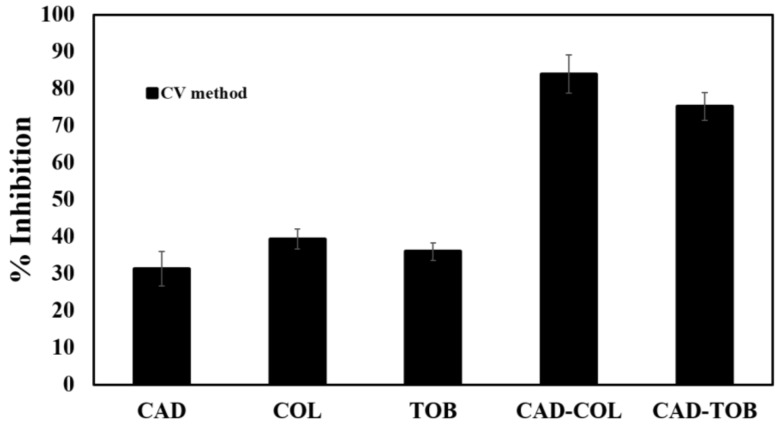
CAD-, colistin (COL)-, TOB-, CAD-COL- and CAD-TOB-mediated inhibition of biofilm formation according to the crystal violet (CV) assay. Data represents the average of six technical replicates from three independent experiments, and error bars indicate ± standard deviation. *p* values of < 0.05.

When combined with 3 mM CAD, the MIC of COL was reduced from 6.8 to 1.7 µM (checkerboard assay). Therefore, to evaluate the combination of CAD and COL on biofilm formation, sub-MICs of 1.5 and 0.9 µM were used for CAD and COL, respectively. Similarly, CAD and TOB were evaluated at 1.5 and 0.9 µM (both sub-MIC values), respectively. CAD showed 31.3% inhibition of biofilm formation, whereas COL and TOB showed approximately 35% inhibition of biofilm formation, as shown by the CV assay (Figure 4). When CAD was combined with COL or TOB, biofilm inhibition was significantly (*p* values of < 0.05) higher at 83.9% and 75.2%, respectively, compared to the untreated control (Figure 4). 

### 3.6. Preformed Biofilm Dispersion by CAD, COL and TOB

**Figure 5:**
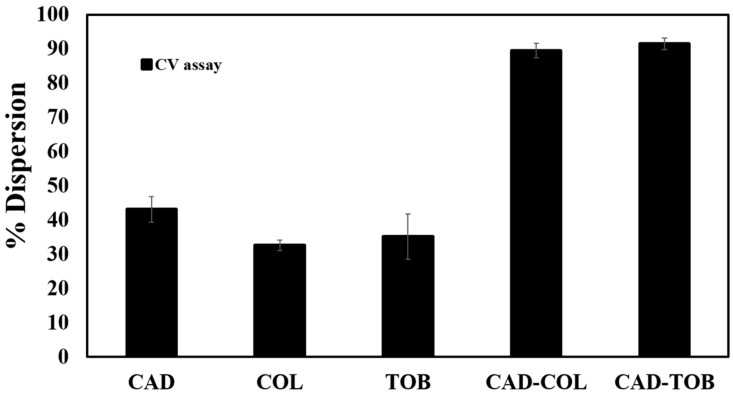
Effect of CAD, TOB, COL, CAD-TOB and CAD-COL on preformed biofilms. Data represent the average of six technical replicates from three independent experiments, and error bars indicate ± standard deviations. *p* values of < 0.05.

To determine if CAD or CAD in combination with COL or TOB can disperse preformed biofilms, biofilms formed on the pegs of a CBD were treated for 3 h and quantified by the CV assay. Treatment with CAD alone showed 43.1% dispersal of the preformed biofilm (Figure 5). 

Similarly, treatment with COL or TOB alone at 0.9 µM concentrations showed 32.6% and 35.2% biofilm dispersion, respectively. When COL or TOB were used in combination with CAD, there was a significant increase (*p* values of < 0.05) in biofilm dispersion as determined by ~90% reduction in preformed biofilm (Figure 5). Thus, CAD combined with COL or TOB synergistically removes established biofilms.

## 4. Discussion

The increase in antibiotic resistance of *P. aeruginosa* and its ability to form persistent biofilms highlight the need to develop alternative clinical treatment strategies [40]. In part, this increased resistance and persistence is associated with biofilm formation [4]. In this study, CAD was combined separately with representatives from four classes of antibiotics (COL, CARB, TOB and ERY), and the synergistic or antagonistic activities towards the MICs of the antibiotics were evaluated with planktonic *P. aeruginosa* PAO1. Only COL showed a synergistic effect with CAD against planktonic cells, although there were slight reductions observed for the combinations of CARB, TOB or ERY with CAD according to the FICI (Table 1). A synergistic activity of CAD and COL was also observed in 10% of clinical *P. aeruginosa* isolates in a prior study [41]. Thus, further work investigating the spectrum of isolates for which COL and CAD show synergistic activity is needed to better clarify this activity. To investigate the possible underlying reason of this synergistic activity, we tested the effect of CAD and COL on biofilm inhibition and also biofilm dispersion assays. Our study on biofilm assays revealed a positive combined effect of CAD and COL at sub-MIC levels which could be due to a disruption in QS system of *P. aeruginosa*. However, this is yet to be tested. 

One issue in the development of antibacterial strategies is the selection for drug resistant mutants, and this concern also applies to QSI-based approaches. Due to the nonlethal nature of QS strategies, it has been suggested that QSI poses no or little selective pressure to pathogens, thus mitigating microbial resistance development to QS inhibitors. However, it was shown that *P. aeruginosa* developed resistance to COL after 15 passages [42]. In this study, serial passage did not lead to a change in the MIC of CAD but was associated with a reduction in fitness after 23 days of daily treatment with CAD (Figure 1). Therefore, based on these results, resistance to CAD was not demonstrated, but some growth fitness reduction occurred. The cytotoxic effect of CAD must also be considered. Previous research demonstrated that 57 mM CAD did not show inhibitory effects on primary human T cells or macrophages [43] or that 4% (303 mM) CAD in calcium carbonate hydrogel had no cytotoxic effect on human gingival fibroblast cells [44]. We evaluated CAD concentrations substantially lower than these (e.g., around the MIC of 11.8 mM), so we predict that our tested CAD concentrations would not be cytotoxic.

In recent years, a number of different antipathogenic drugs and strategies have been developed to reduce bacterial virulence by disrupting QS [45]. Thus, blocking QS in *P. aeruginosa* by QSI is suggested as a promising strategy for the treatment of infections [20,46,47]. It was recently shown that iberin, from horseradish, has QSI activity in *P. aeruginosa* according to two reporter systems tested: *lasB::gfp* and *rhlA::gfp* [15]. Cinnamon oil (in which CAD is one of the major ingredients) has been previously reported to show QSI effect on *P. aeruginosa* [48]. This study demonstrated a reduction in long-chain acyl-homoserine lactones (AHLs) and pyocyanin of the QS system by cinnamon oil. In our study, CAD inhibited the Las-, Rhl- and PQS-mediated expression of GFP in a concentration-dependent manner, without affecting microbial growth. Similar results with CAD significantly reducing the expression of LasR and RhlR were reported [28]. However, there was no demonstrated inhibition of CAD on the PQS system of *P. aeruginosa* [28]. PQS regulates the release of extracellular DNA, which is an important structural component of *P. aeruginosa* biofilms [33]. Other previous studies found that CAD inhibited pyocyanin production [25,48,49,50]. A study of CAD, QS and pyocyanin demonstrated that CAD specifically targets the short-chain AHL synthase (RhlI) [50], which is important for pyocyanin production. Thus, our work expands the spectrum of CAD activity beyond its inhibition of pyocyanin production by showing that CAD is inhibitory towards the Las and Rhl systems, and additionally towards PQS system of *P. aeruginosa*, in a concentration-dependent manner (Figure 2d).

We also confirmed the QSI activity of TOB, which is consistent with previously published findings [39]. TOB did not affect the transcription rates of the *lasI* or *rhlI* (for AHL synthases), but there was a substantial reduction (20–25%) in short-chain AHL when *P. aeruginosa* PUPa3 was grown in the presence of sub-MIC TOB [39]. However, in our study, CAD and TOB reduced the expression of *lasB, rhlA* and *pqsA* in a LasR-independent manner. TOB alone reduced RhlR-controlled GFP expression by 32.2%, and the combination of CAD-TOB repressed GFP by 64.7%. Similar results were observed for LasB and PQS (Figure 3d). Thus, sub-MIC CAD and TOB combined is an avenue that should be explored to increase the chance of success in the treatment of *P. aeruginosa* infections as it does not kill the bacteria, which limits its tendency to develop resistance to CAD-TOB. Instead of imposing direct selective pressure on the growth of *P. aeruginosa*, CAD and TOB could preferentially reduce QS-based communication and eventually attenuate cascades of gene expression and production of virulence factors which could lead to less pathogenicity. It was also demonstrated that they have a combined efficacy in biofilm formation inhibition and dispersion of preformed biofilms by disrupting QS. 

Given that biofilm formation in *P. aeruginosa* is partially QS controlled, COL and TOB were investigated for their control of biofilms in combination with CAD. Previously, CAD demonstrated a significant reduction in biofilm formation at sub-MIC level, and CAD at 3 mM (sub-MIC) was also able to disperse preformed biofilm up to 95%, confirmed by confocal laser scanning microscopy images [26]. As predicted, a substantial inhibition in biofilm formation was observed when CAD was combined with either COL (83.9%) or TOB (75.2%) compared to the CAD-treated (31.3%) biofilms alone (Figure 4). Thus, we propose that a combination treatment could be aimed first at disabling the QS system and then inhibiting biofilm formation. This could be a promising strategy to prevent biofilm infections from developing into the chronic state. For example, it was reported that treatment with a QS inhibitor and TOB resulted in an increased clearance of *P. aeruginosa* in a foreign body infection model, demonstrating that this approach can work in vivo [23]. Another study demonstrated eradication of *P. aeruginosa* biofilms when a QS inhibitor and TOB were combined [51]. Sub-MIC CAD and 2×MIC of TOB had demonstrated a significant inhibition of *P. aeruginosa* biofilms [22]. We focused on sub-MICs of CAD and COL or TOB, as our main focus was to control the signaling pathways of *P. aeruginosa* in attempts to avoid antimicrobial resistance. CAD in combination with COL or TOB dispersed preformed biofilms by up to ~90% compared to untreated biofilms, whereas COL and TOB alone only dispersed biofilms by 32.6% and 35.2%, respectively (Figure 5). These findings could be due to the absence of functional QS as CAD is able to disrupt the QS systems, thus making *P. aeruginosa* more susceptible to antibiotics and showing synergistic effects on inhibiting biofilms and dispersing preformed biofilms.

In conclusion, CAD and COL showed a synergistic action in killing planktonic cells of *P. aeruginosa*. The 23 passages with CAD reduced *P. aeruginosa* growth rate, but these levels of passaging are beyond what might occur in a clinical setting. CAD is a QS inhibitor at sub-MICs, and the additive effect of TOB with CAD was also apparent in the inhibition of QS. CAD combined with COL or TOB reduced biofilm formation and increased dispersal of preformed biofilm cells compared to individual treatment. Collectively, these results demonstrated that CAD as a QS inhibitor may increase the success of antibiotic treatment in combination therapy by disrupting QS more efficiently and subsequently increasing the susceptibility of bacterial biofilms to antibiotic therapy. Thus, CAD shows promise for use as an antipathogenic compound that is enhanced if used in combination with antibiotics. 

## Figures and Tables

**Table 1:** MICs of antibiotics alone and in combination with cinnamaldehyde (CAD).

Antibiotic	MIC ^1^		FICI ^2^	Activity
	Alone	Combined with 3 mM CAD		
Colistin	6.8 μM	1.7 μM	0.5	Synergistic
Carbenicillin	338.3 μM	169.2 μM	0.75	Indifferent
Tobramycin	3.3 μM	1.7 μM	0.75	Indifferent
Erythromycin	348.8 μM	174.4 μM	0.75	Indifferent

^1^, MIC—minimum inhibitory concentration. ^2^, FICI— fractional inhibitory concentration index. The MIC of COL was 6.8 µM, which was reduced to 1.7 µM when combined with CAD. The calculated FICI for COL plus CAD was 0.5, which indicates synergistic activity. Although carbenicillin (CARB), tobramycin (TOB) and erythromycin (ERY) showed reduced MICs when combined with CAD (Table 1), their FICI values of 0.75 deemed them to be indifferent or having no synergy with CAD.
